# Adenomyomatous Hyperplasia of Ampulla of Vater and a Concomitant Renal Tumor: A Case Report

**DOI:** 10.7759/cureus.20258

**Published:** 2021-12-08

**Authors:** Luisa Frutuoso, Ana Marta Pereira, Lucia Carvalho, Gil Gonçalves, Mário Nora

**Affiliations:** 1 General Surgery, Centro Hospitalar de Entre Douro e Vouga, Santa Maria da Feira, PRT; 2 General Surgery, Champalimaud Foudation, Lisbon, PRT; 3 General Surgery, Centro Hospitalar Entre o Douro e Vouga, Santa Maria da Feira, PRT

**Keywords:** pancreas, pancreatodudenectomy, renal neoplasms, ampulla of vater, adenomyomatous hyperplasia

## Abstract

Adenomyomatous hyperplasia is an extremely rare lesion encountered in the ampulla of Vater. Less than 50 cases have been described, most of them with clinical consequences of biliary obstruction, misdiagnosing it as a malignancy. The authors present a concomitant case with a renal tumor, its diagnosis, management, and clinical relevance, as well as a brief revision of the literature. Ampullar and renal tumors were found in a 74-year-old female, in the imagiologic study of a low back pain, in the emergency department. Both were considered malign after further study, and pancreatoduodenectomy with partial nephrectomy was proposed. There is no accurate diagnostic tool to differentiate the benign nature of adenomyomatous hyperplasia and extensive operations are often performed. As an incidental finding in the study of another tumor, this case raises the concern about which and how to treat both tumors, taking into account the morbidity of the respective interventions.

## Introduction

Benign tumors constitute 6% of all extrahepatic biliary tree neoplasms and are responsible for 0.1% of all biliary tract operations [1]. Adenomyomatous hyperplasia (AH) or adenomyomiosis can occur anywhere in the gastrointestinal tract. It is an extremely rare benign lesion of the extrahepatic biliary tree, more often encountered in the gallbladder, but only a few cases have been described in the ampulla of Vater [2-4].

The histopathogenesis and natural history of these lesions in the common bile duct (CBD), including the ampulla of Vater, is not clear and malignant transformation in other organs as well as recurrence after local resection has been described [1,5]. Diagnostic tools, including endoscopy with ultrasonography and biopsy, are frequently not accurate enough to rule out a malignancy, and these diagnostic difficulties have led to extensive operations. The appropriate management of these tumors is not established.

Unlike the other counterparts in the gastrointestinal tract, the ampulla of Vater´s tumors is responsible for the biliary obstruction, which leads to significant clinical consequences. Jaundice and abdominal pain are the main complaints of these patients although an incidental finding in the study of another neoplasm has been described as well [2]. The latter adds a dilemma about how and when to treat each tumor taking into account both are suspicious of malignancies.

We report a case of a patient AH of the ampulla of Vater and a concomitant renal tumor, its diagnosis, management, and clinical relevance, as well as a brief revision of literature.

## Case presentation

A 74-year-old female, with a past history of renal lithiasis and lumbar hernia, recurred to the emergency department (ED) complaining of acute low back and abdominal pain. An abdominopelvic CT scan in the ED revealed not only the main cause of the pain, calculous hydronephrosis but also two incidentally distinct lesions: a left renal tumor with malignant features and a 15-mm nodular lesion conditioning CBD and wirsung ectasia. No hepatic changes or cholestasis were observed in the laboratory study. Medical treatment for ureterolithiasis was done with complete symptomatic resolution.

Further study was done for both tumors. Laboratory tests showed normal hepatic and renal function, with no cholestasis. Serum carcinoembryonic carcinogen and carbohydrate antigen levels were not increased.

**Figure 1:**
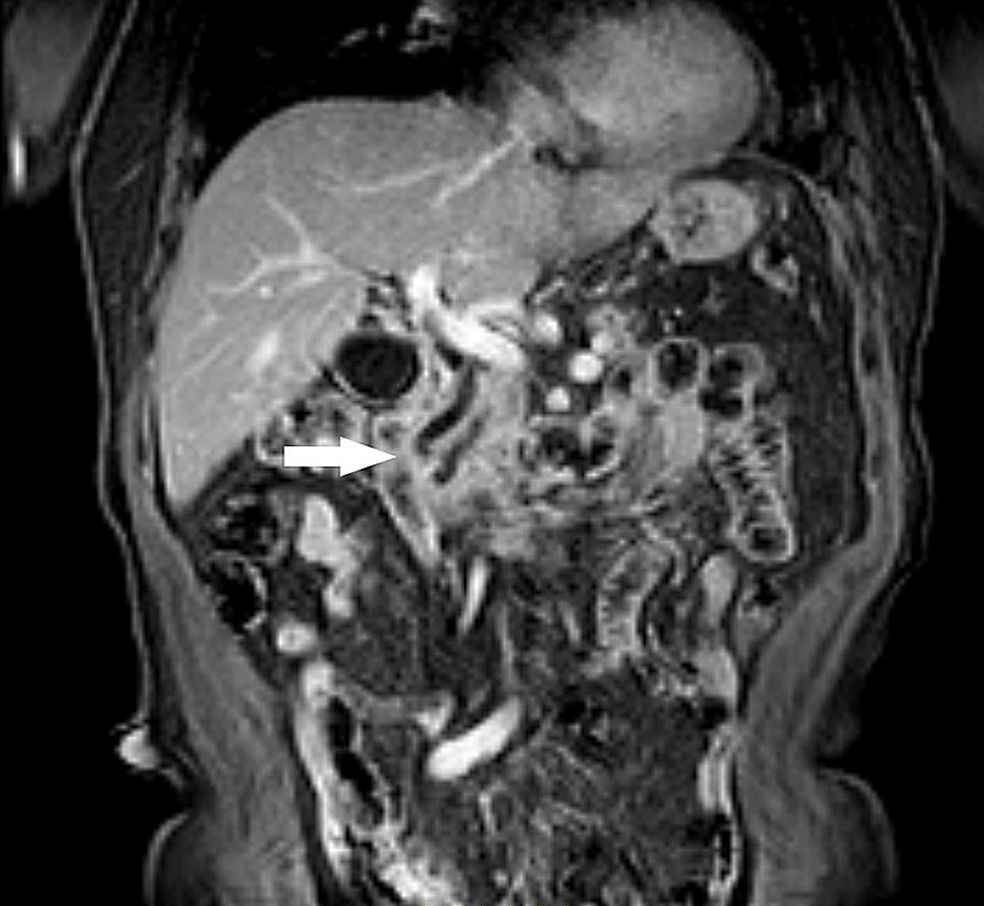
MRCP showed bicanalar dilation with abrupt stenosis MRCP - magnetic resonance cholangiopancreatography

A magnetic resonance cholangiopancreatography (MRCP) confirmed bicanalar dilation (CBD and wirsung) with abrupt stenosis at the ending of both channels, although no evident nodular lesion, as well the renal tumor (Figure 1). Fluorodeoxyglucose positron emission tomography-computed tomography scan (PET-FDG/CT) was inconclusive.

Endoscopic ultrasound was attempted and no ampullar changes were demonstrated, but a cystic lesion was apparent at the terminus of the CBD (choledococele), so biopsies were not taken.

**Figure 2:**
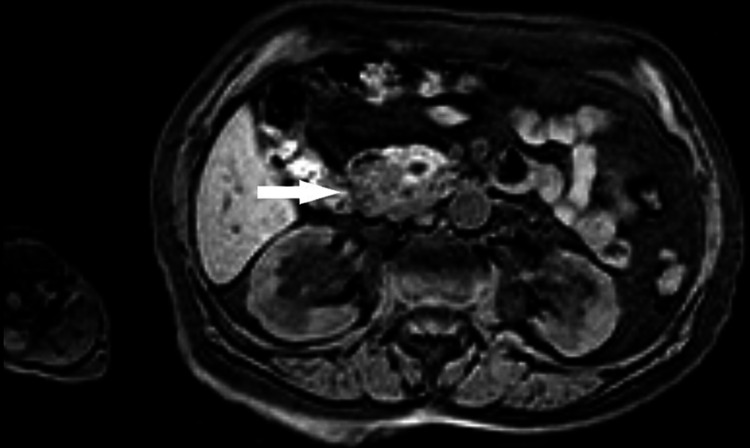
MRCP showed an ampullar mass

Three months later, an MRCP was repeated which revealed at this time an ampullar mass causing bicanalar obstruction, and so, a CBD malignancy could not be ruled out (Figure 2).

A multidisciplinary team discussed the case and the patient was proposed to pylorus-preserving pancreatoduodenectomy in combination with partial nephrectomy, in the same surgical time, to which the patient consented.

Macroscopic examination of the surgical specimen revealed a polypoid lesion in the ampullar region with 1.2 cm.

Pathologic examination demonstrated chronic polypoid fibrous ampullitis with glandular AH. No malignant cells were identified. The partial nephrectomy specimen revealed a clear cell renal carcinoma (pT1aNxVx).

The patient's condition was complicated with postoperative intra-abdominal abscess followed by surgical drainage. Full recovery and discharge were possible one month after surgery.

Eight months later, the patient is doing well, with no complaints or recurrence of both lesions.

## Discussion

AH is an extremely rare lesion encountered in the ampulla of Vater and can be misdiagnosed as an ampullar adenoma or carcinoma. To our knowledge, less than 50 cases have been described in the indexed English literature [6]. This is the second case in our institution [4].

It is difficult to determine the real incidence, but a frequency of 0.13% has been reported in a series of consecutive 3,131 endoscopic retrograde cholangiograms (four cases) [7]. Higher frequency was noted in a post-mortem specimens study, in which 54% of 100 unselected patients had this lesion, with no clinical significance [8]. Another interesting observation of this study was the presence of other concomitants, more important findings, apparently not correlated with the AH, such as thyroid adenomas, gastric, colonic and uterine polyps, and even adenocarcinomas of the stomach, colon, and rectum, although no constant association was observed. This could bring up the hypothesis of the AH as a sentinel lesion for other tumors, giving this case as an example, once the renal tumor was the main concern of this patient´s condition.

Histologically, AH is defined as a nodular lesion or a mucosal thickening with a proliferation of smooth muscle and epithelial components, as well as glandular hyperplasia without cellular atypia. Its histogenesis is not clear and many theories have been proposed. Incomplete heterotopic pancreas (type III according to Heinrich classification) seems to be the most widely accepted theory, although some authors defend that this lesion may have an inflammatory origin [9,10]. Baggenstoss also highlights the fact that the papillar region as a transitional epithelial zone, is susceptible to local irritation inducing polypoid hyperplasia [11]. This case represents a glandular AH associated with a chronic inflammatory polypoid ampullar lesion, which goes with this hypothesis.

The preoperative diagnose is challenging and imaging is not accurate enough to distinguish an AH from adenoma or carcinoma. A definitive diagnose is only possible with histological examination of the completed resected mass [2,12,13]. Preoperative biopsies are often useless once the absence of malignant features cannot exclude malignancy. In a series of 13 patients with AH of the ampulla of Vater, preoperative papillar biopsies showed epithelial cell atypias, suggesting dysplasia in 66.6% of the cases [2]. A normal appearance of the papilla, as in this case, with an ampullar non-protruding type lesion, turns the endoscopic investigation even more difficult [14].

Despite AH is considered a slow-growing benign lesion, in this case, rapid growth was observed during the investigation, with the development of an ampullar mass noted in the second MRCP, three months after the first with no visible lesion. A similar case was described by Ulich et al, who suggest that some of these lesions might be truly neoplastic in nature, in accordance with some authors that consider the AH of the ampulla of Vater a premalignant lesion [11,15].

There is no widely accepted approach to managing these patients. Ampullectomy, local resections with endoscopic snare excisions, or simply biliary tree drainage with sphincterotomy have been offered when a preoperative diagnosis is possible, avoiding postoperative morbidity and mortality, which is not neglectable in a pancreaticoduodenectomy. Sphincterotomy was unsatisfactory in previous cases in severely symptomatic patients or showed recurrence [2]. Extensive surgeries have also been recommended by some authors taking into account a recurrence rate of 22% for local resection of benign tumors of extrahepatic biliary duct or ampulla of Vater [1].

This particular case raises a dilemma: both tumors are suspicious of malignancy, being an ampullar tumor more aggressive; in spite of the good physical condition of the patient for the age, the physiological status is limited, and the decision to operate involves substantial risk. Nevertheless, radical surgery is still the chosen approach when an ampullar carcinoma cannot be ruled out, as in this case. Further studies have to be done to improve the diagnostic accuracy and provide less aggressive interventions.

## Conclusions

Adenomyomatous hyperplasia of the ampulla of Vater is an extremely rare tumor of the biliary tree. Unlike in other organs, this location has important clinical consequences, being misdiagnosed as an adenoma or carcinoma in most of the cases. There is no accurate method to distinguish these lesions from an adenoma or carcinoma, and so, extensive surgery is frequently adopted.

Efforts must be done to improve the diagnostic tools. Cases with concomitant neoplasms are not negligible in the literature, with decision-making implications and increased surgical risk.
